# Integrating High-Performance Flexible Wires with Strain Sensors for Wearable Human Motion Detection

**DOI:** 10.3390/s24154795

**Published:** 2024-07-24

**Authors:** Pucheng Wu, Hu He

**Affiliations:** State Key Laboratory of Precision Manufacturing for Extreme Service Performance, College of Mechanical and Electrical Engineering, Central South University, Changsha 410083, China; hnlgwpc@163.com

**Keywords:** flexible electronics, liquid metal, conductive ink, sintering process, mechanical stability

## Abstract

Flexible electronics have revolutionized the field by overcoming the rigid limitations of traditional devices, offering superior flexibility and adaptability. Conductive ink performance is crucial, directly impacting the stability of flexible electronics. While metal filler-based inks exhibit excellent conductivity, they often lack mechanical stability. To address this challenge, we present a novel conductive ink utilizing a ternary composite filler system: liquid metal and two micron-sized silver morphologies (particles and flakes). We systematically investigated the influence of filler type, mass ratio, and sintering process parameters on the composite ink’s conductivity and mechanical stability. Our results demonstrate that flexible wires fabricated with the liquid metal/micron silver particle/micron silver flake composite filler exhibit remarkable conductivity and exceptional bending stability. Interestingly, increasing the liquid metal content results in a trade-off, compromising conductivity while enhancing mechanical performance. After enduring 5000 bending cycles, the resistance change in wires formulated with a 4:1 mass ratio of micron silver particles to flakes is only half that of wires with a 1:1 ratio. This study further investigates the mechanism governing resistance variations during flexible wire bending. Additionally, we observed a positive correlation between sintering temperature and pressure with the conductivity of flexible wires. The significance of the sintering parameters on conductivity follows a descending order: sintering temperature, sintering pressure, and sintering time. Finally, we demonstrate the practical application of this technology by integrating the composite ink-based flexible wires with conductive polymer-based strain sensors. This combination successfully achieved the detection of human movements, including finger and wrist bending.

## 1. Introduction

In recent years, with advancements in science and technology and the improvement of living standards, flexible electronics have garnered broad application prospects due to their flexibility, thinness, and bendability. They are increasingly used in wearable devices [1,2], medical diagnosis [3,4], health monitoring [5,6], and electronic skin [7,8]. Conductive ink has become a research hotspot in the field of flexible electronics because of its excellent flexibility, printability, and conductive properties [9]. Conductive ink can be directly printed onto a flexible substrate, allowing for the one-step deposition and patterning of functional materials, thereby constructing flexible circuits.

The current research on conductive inks primarily focuses on carbon-based conductive inks [10,11], metal-based conductive inks [12,13], and composite conductive inks [14,15]. Metal-based conductive inks are particularly favored due to their superior conductivity and widespread application. These inks are typically prepared using metal fillers such as gold, silver, and copper. However, the high cost of gold and the susceptibility of copper to oxidation, which reduces its conductivity, limit their practical applications. Consequently, silver-based conductive inks are the most widely used and offer the best performance. Mo et al. [16] successfully synthesized silver nanoparticles with an average radius of 48–176 nm by adjusting the silver ion concentration during the reaction process and used them to prepare conductive inks. When maintained at 140 °C for 10 min, the printed pattern exhibited an optimal resistivity of 4.6 μΩ·cm, which is very close to that of bulk silver (1.58 μΩ·cm). They also conducted bending tests on silver electrodes printed on coated paper. The results showed robust responses, with resistance change rates of 8.01% and 18.55% for samples with bending radii of 2.5 mm and 1.0 mm, respectively, after 1000 bending cycles. Maeda et al. [17] prepared a conductive ink containing micron-sized silver particles and silver flakes and printed the ink on PEN film. They found that, although the resistivity of the material increased by 3.7 times when the content of micron flakes (Ag-MFS) increased from 0 to 30 wt%, the bending stability of the conductive pattern improved, because Ag-MFS inhibited crack formation under cyclic bending stress. Zhao et al. [18] developed a conductive ink comprising micron-sized silver flakes and nano-sized silver particles, with silver flake diameters of 1–3 μm and nanoparticle sizes of 20–50 nm. The printed conductive pattern achieved a resistivity of 10.8 μΩ·cm after being maintained at 140 °C and 10 MPa pressure for 40 min. After 6000 bending cycles, the resistivity of the silver pattern, sintered for 20 min, did not change significantly. Liquid metal (LM) has also attracted extensive research interest in the field of conductive inks due to its high conductivity and fluidity. Although liquid metal conductive ink exhibits excellent conductivity and mechanical stability, it usually requires additional circuit activation due to the presence of an outer layer of gallium oxide film. Wu et al. [19] prepared LM-LAPONITES (LML) ink using special silicate flakes (Laponite) and LM. During ink evaporation, Laponite generates capillary forces, which can sinter LM droplets and activate the conductivity of the conductive pattern. Liu et al. [20] proposed an ultrasonic sintering strategy for patterned LM circuits, enabling the rapid production of conductive LM circuits on both rigid and flexible substrates. Compared to mechanically sintered conductive circuits, ultrasonic sintering preserves the shape of the LM ink pattern and can be achieved on rough or grooved surfaces.

Although the printed patterns prepared by silver-based conductive ink exhibit strong conductivity, their electrical stability is relatively weak. Liquid metal is a metallic material that remains in a liquid state at room temperature and possesses high conductivity, making it an ideal filler for preparing conductive inks. However, due to the presence of an outer layer of gallium oxide film, the conductive patterns prepared by liquid metal conductive ink, while demonstrating strong conductivity and electrical stability, require additional processes to activate their conductivity, which are complex and unstable. Therefore, combining liquid metal with silver filler as a conductive filler represents a novel approach to the study of conductive inks.

In this study, we utilized liquid metal and two different forms of silver fillers as conductive fillers to prepare liquid metal/silver conductive inks doped with varying forms of silver fillers. We then used these conductive inks to fabricate conductive circuits. We investigated the effects of the types of added silver fillers on the conductivity and mechanical stability of the conductive circuits and elucidated their bending mechanisms. Additionally, we studied the impact of the mass ratio of conductive fillers on the conductive properties and mechanical stability of the conductive inks. Furthermore, we examined the influence of the sintering process on the conductive properties of these inks. Finally, we integrated the flexible wires based on conductive ink with the strain sensor based on conductive polymer composites to verify its potential application in the field of flexible electronics. Through these studies, we aim to identify a method for preparing conductive inks that exhibit both high conductivity and excellent mechanical stability, thereby providing technical support for future applications in the field of flexible electronics.

## 2. Materials and Methods

Galinstan liquid metal (LM) was obtained from Beijing Mengzhimo Technology Co., Ltd. (Beijing, China). Silver fillers, including micron particles (2 μm), flakes (1–3 μm, 100–200 nm thick), and nanoscale particles (20 nm), were purchased from Shanghai MacLean Biochemical Technology Co., Ltd. (Shanghai, China) and Beijing Zhongke Keyou Technology Co., Ltd. (Beijing, China), respectively. Carbon nanotubes (10–20 nm outer diameter, 10–30 μm length) were supplied by Chengdu Zhongke Times Nano Energy Technology Co., Ltd. (Chengdu, China) Solvents (anhydrous ethanol, ethylene glycol, and n-hexane) and polyvinylpyrrolidone (PVP K90) were procured from Shanghai MacLean Biochemical Technology Co., Ltd. (Shanghai, China) and Hefei BASF Bio Co., Ltd. (Hefei, China), respectively. Deionized water was generated in the laboratory. For device fabrication, polydimethylsiloxane (PDMS, Sylgard 184) was purchased from Dow Corning Corporation (Midland, MI, USA), photosensitive resin from Shenzhen Hesu Photocuring Materials Co., Ltd. (Shenzhen, China), release agent from Wuxi Fites Electronic Technology Co., Ltd. (Wuxi, China), conductive silver paste from Shenzhen Saiya Electronic Paste Co., Ltd. (Shenzhen, China), polyimide (PI) tape (55 μm thick) from Hangzhou Youbisheng Tape Co., Ltd. (Hangzhou, China), and PI film (0.1 mm thick) from Shenzhen Beilong Electronic Materials Co., Ltd. (Shenzhen, China).

**Figure 1:**
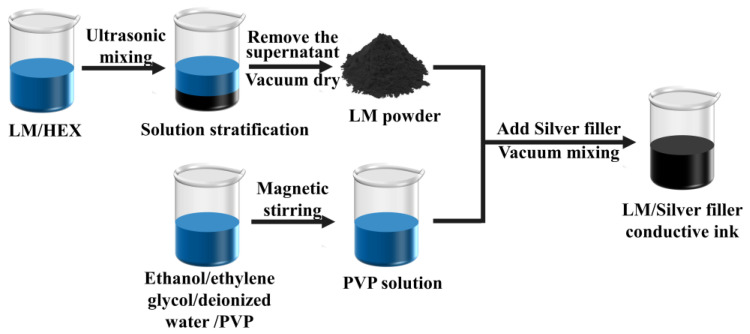
Preparation process of liquid metal/silver filler conductive ink.

As shown in Figure 1, liquid metal (LM, 4 g) was dispersed in n-hexane (HEX, 20 g) using ultrasonic treatment (FS-1200N, Shanghai Shengxi Ultrasonic Instrument Co., Ltd. (Shanghai, China)) at 45% maximum power (1200 W) for 50 min. This treatment precipitated LM particles, which were then separated by removing the supernatant. The precipitate was vacuum-dried at 100 °C for 1 h (DZF-6050, Shanghai Yiheng Scientific Instrument Co., Ltd. (Shanghai, China)) to remove the residual n-hexane, yielding LM powder. A mixture of anhydrous ethanol (5 g), ethylene glycol (2 g), and deionized water (3 g) was stirred (MS5, Qun’an Experimental Instrument Co., Ltd. (Zhejiang, China)) until homogenous. Then, 400 mg of PVP was added and continuously stirred until dissolved, forming a PVP solution. LM, silver filler, and the PVP solution were mixed in a predetermined mass ratio. The mixture underwent vacuum degassing (TMV-310TT, Shenzhen Simaida Electronics Co., Ltd. (Shenzhen, China)) for 5 min to eliminate bubbles, resulting in a 50 wt% conductive ink.

A 30 mm long, 0.055 mm thick hollow polyimide (PI) tape was used as a mask for the conductive ink. It was adhered to a PI film, defining the desired conductive line pattern. The conductive ink was then applied liberally to the exposed area within the hollow PI tape. A flat scraper evenly distributed the ink, and excess material was removed. Subsequently, the assembly was dried at 60 °C for 30 min on a heating table to solidify the ink. After drying, the PI tape mask was peeled off, revealing the patterned conductive line. Thermal sintering of the conductive line employed a microcomputer-controlled heating table with variable temperature and time settings. For hot pressing sintering, a custom-built device applied pressure while heating, ensuring the integrity of the conductive line during the process. A PI film layer protected the pattern during hot pressing.

**Figure 2:**
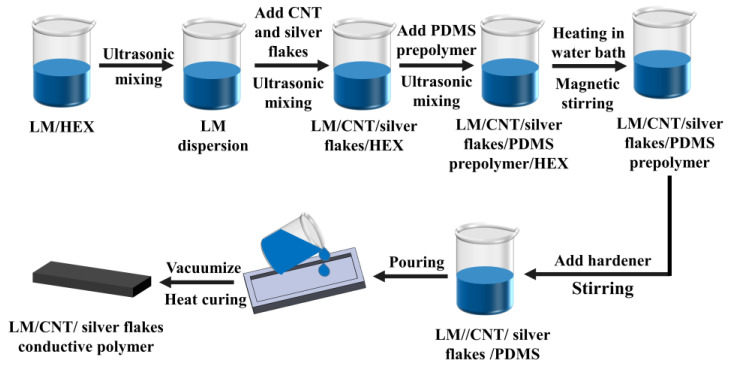
Schematic diagram of the preparation process of a conductive polymer.

Following our previous work [21,22], as shown in Figure 2, liquid metal was first dispersed in n-hexane using ultrasonication (30 min, 45% maximum power, 1200 W) to create a homogenous mixture. Subsequently, carbon nanotubes (CNTs) and silver flakes were added and sonicated for 5 min (20% maximum power) to achieve uniform distribution within the n-hexane. PDMS prepolymer was then incorporated and sonicated for another 5 min (20% maximum power) for further dispersion. Following sonication, the mixture was stirred at 60 °C in a magnetic stirrer (details omitted) to evaporate n-hexane. After complete n-hexane removal, a curing agent (10:1 mass ratio with a PDMS prepolymer) was added and manually stirred for even distribution. Finally, the mixture was degassed in a vacuum oven (20 min) and cured at 120 °C for 1 h, yielding the final LM/CNTs/silver flakes conductive polymer.

A high-precision digital bridge (TH2810B+, Changzhou Tonghui Electronic Co., Ltd. (Changzhou, China)) measured the resistance between both ends of the conductive lines. Mechanical stability was evaluated using an electronic universal testing machine (UTM2502, Shenzhen Sansi Zongheng Technology Co., Ltd. (Shenzhen, China)) and a micro-force fatigue testing machine (ElectroForce 3200, TA Instruments, New Castle, DE, USA). Both machines secured the samples and controlled their outward bending for static and dynamic stability tests, respectively. A high-precision digital bridge monitored the corresponding electrical signals during bending. Notably, the bending radius estimation method was adopted from References [23,24]. Based on our laboratory testing conditions, we set the movement distance of the motion platform to 2 to 10 mm, and combined with our demonstration of human joint motion monitoring application, the bending radius of the flexible wires was within the range of our study. The microstructure of conductive patterns fabricated with different inks was examined using a scanning electron microscope (SEM) (VEGA3, Tescan (China) Co., Ltd. (Shanghai, China)).

## 3. Results and Discussion

### 3.1. Influence of Conductive Filler Combinations on the Performance of Conductive Inks

**Figure 3:**
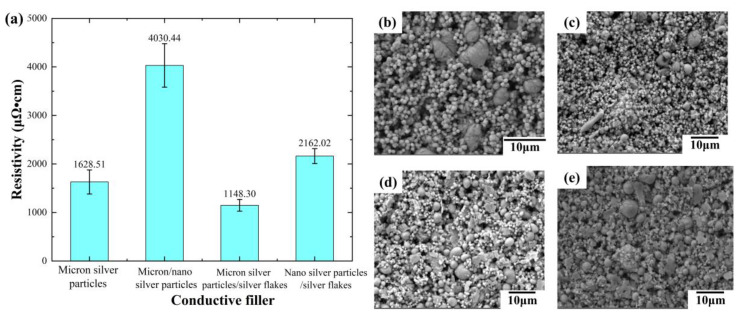
Initial resistivity and SEM images of conductive lines prepared with different filler inks: (**a**) initial resistivity of different conductive lines; (**b**–**e**) SEM images of different conductive lines; (**b**) liquid metal/micron silver particles; (**c**) liquid metal/micron silver particles/silver nanoparticles; (**d**) liquid metal/micron silver particles/silver flakes; (**e**) liquid metal/silver nanoparticles/silver flakes (the mass ratio of liquid metal to silver fillers is 1:1, and the mass ratio of different silver fillers is 1:1).

Four conductive inks were formulated by varying the type of silver filler, and their initial conductivity after pattern formation was measured. As shown in Figure 3a, the ink containing liquid metal, micron silver particles, and silver flakes exhibits the superior initial conductivity (1148.30 μΩ·cm) without hot pressing. This surpasses the conductivity of the ink with only liquid metal and micron silver particles, suggesting that silver flakes enhance the electrical performance. Conversely, the inclusion of silver nanoparticles significantly reduces conductivity. The resistivity of the ink containing all three fillers (4030.44 μΩ·cm) is more than double that without nanoparticles.

Conductive ink performance is heavily influenced by the content and characteristics (morphology and particle size) of the fillers. When solely relying on micron-sized silver particles for conductivity (no doping with different-sized fillers), their contact mode is primarily point-to-point, as illustrated in Figure 3b. However, incorporating silver flakes facilitates a transition from point contact to partial surface or line contact (Figure 3d), ultimately improving electrical conductivity.

In contrast, the small size and agglomeration tendency of silver nanoparticles hinder their dispersion within the ink, consequently impeding the formation of a conductive path. This phenomenon, visualized in Figure 3c,e, leads to a decrease in overall conductivity. Therefore, the addition of silver nanoparticles in this case has a detrimental effect.

**Figure 4:**
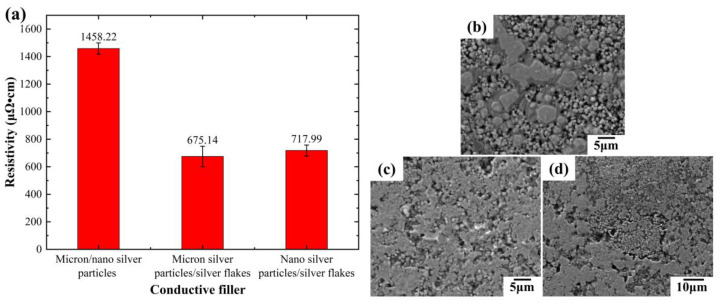
Electrical properties and SEM images of conductive lines prepared with different filler inks after hot pressing sintering: (**a**) electrical properties of different conductive lines after hot pressing sintering; (**b**–**d**) SEM images of different conductive lines after hot pressing sintering: (**b**) liquid metal/micron silver particles/silver nanoparticles; (**c**) liquid metal/micron silver particles/silver flakes; (**d**) liquid metal/silver nanoparticles/silver flakes (the mass ratio of liquid metal to silver fillers is 1:1, and the mass ratio of different silver fillers is 1:1).

The electrical properties were then measured for the remaining three conductive lines, each containing different silver fillers, after hot pressing and sintering at 210 °C and 2.5 MPa for 20 min (Figure 4a). Hot pressing and sintering significantly improved the electrical conductivity of all the lines. Compared to the untreated state, the resistivity of the line containing liquid metal, micron silver particles, and silver nanoparticles decreased from 4030.44 μΩ·cm to 1458.22 μΩ·cm, representing nearly a twofold increase. Similar improvements were observed for lines containing other filler combinations. To further investigate this effect, we examined the hot-pressed conductive lines using a scanning electron microscope (SEM). Compared to untreated lines, the hot-pressed lines exhibited smoother surfaces with significantly reduced surface pores and improved contact between conductive fillers (Figure 4a). These changes directly contributed to the enhanced electrical properties.

Interestingly, for the line containing liquid metal, micron silver particles, and silver nanoparticles, block-like features were observed on the surface after hot pressing (Figure 4b). We hypothesize that this is due to the melting of silver nanoparticles and the formation of sintering necks under the applied pressure and temperature (210 °C, 2.5 MPa). Additionally, the pressure likely disrupts the oxide film on the liquid metal, causing spilled liquid metal to connect the sintering necks. This unique micromorphology significantly improves the electrical conductivity. Energy-dispersive X-ray spectroscopy (EDS) analysis of these surface blocks revealed their primary composition as Ag elements, with the connecting parts containing Ga, In, Sn, and other elements from the liquid metal, supporting our hypothesis. Notably, this phenomenon was not observed in lines containing silver flakes (Figure 4c,d). Under pressure, the silver flakes likely intermix with the liquid metal, leading to a smoother and more compact surface structure, ultimately enhancing conductivity.

**Figure 5:**
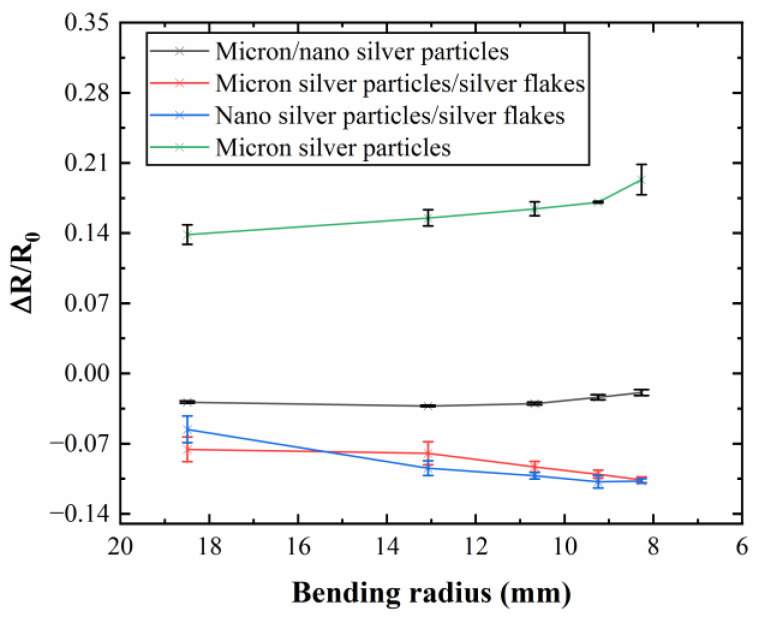
Resistance change rate of conductive lines prepared by different conductive inks under different bending radii.

Following the conductivity test of the conductive line, the bending radius and bending cycle tests were conducted to study the mechanical stability of the conductive network with different combinations of silver fillers. In Figure 5, it can be observed that the mechanical properties of silver fillers with different characteristic parameters are superior to those of single-micron silver fillers. Typically, with an increase in the degree of bending, the resistance of the conductive line increases, indicating that a smaller bending radius leads to greater resistance in the conductive line due to the formation of microcracks under bending stress. However, in Figure 5, an opposite trend is noted, where the resistance of the conductive line decreases with an increase in the degree of bending. This phenomenon occurs because, during bending, the liquid metal in the conductive network breaks, resulting in the outflow of the internal liquid metal, which mitigates the effects of microcracks caused by the bending of the conductive network. Simultaneously, there is a dynamic process of reconstruction and destruction of the conductive network. During bending, these processes compete: if the reconstruction rate exceeds the failure rate of the conductive network, the resistance of the conductive line decreases; otherwise, it increases.

Voids within the network can contribute to crack formation. Adding silver nanoparticles fills these voids, leading to lower resistance at smaller bending angles, although resistance might increase beyond a certain threshold. The inclusion of silver flakes further enhances bending stability, as shown by the lower resistance change rate compared to formulations lacking them (Figure 5). This effect is likely due to the increased number of liquid metal particles, promoting faster network reconstruction relative to failure. Notably, conductive ink containing a combination of liquid metal, micron silver particles, and silver flakes exhibits a decreasing resistance change rate with the increasing bending angle (Figure 5), indicating exceptional mechanical stability. At maximum bending, resistance even decreases by 10.61%.

Conductive lines containing various conductive fillers were subjected to 5000 bending cycles, with resistance changes monitored throughout and after the test. Compared to lines filled solely with micron silver particles, those incorporating silver fillers with different characteristics exhibited enhanced mechanical stability and bending durability.

Repetitive mechanical strain can induce microcracks within the conductive network [16,18,25], compromising its continuity and potentially leading to network failure. However, this strain can also rupture the outer oxide film of the liquid metal, allowing the internal liquid metal to flow and repair the microcracks, ultimately facilitating network reconstruction. Additionally, incorporated silver nanoparticles (AgNPs) act as bridges between adjacent conductive fillers, increasing contact points and enhancing network stability. The larger surface area of silver flakes makes the liquid metal more susceptible to rupture under strain, resulting in more broken liquid metal connections and further improving network stability.

**Figure 6:**
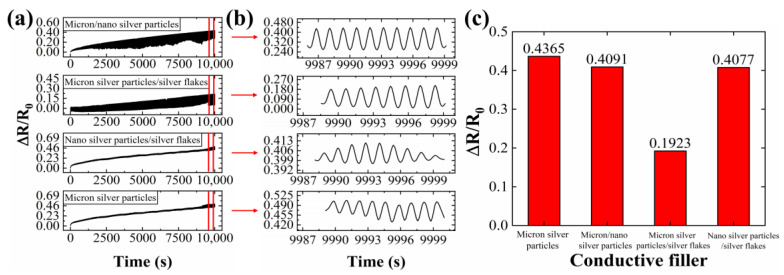
The resistance change rate of conductive lines prepared by different conductive inks under 5000 bending cycles: (**a**) the resistance change rate during 5000 bending cycles; (**b**) the magnification of the red area in figure (**a**); (**c**) the resistance change rate after 5000 bending cycles.

As shown in Figure 6c, the resistance change rate of the conductive line containing a combination of liquid metal, micron silver particles, and silver flakes is significantly lower compared to lines with only micron silver particles. The optimal combination of conductive fillers (LM/micron silver particles/silver flakes) exhibited a resistance change rate of only 19.23% after 5000 bending cycles, demonstrating the excellent mechanical stability and bending resistance of this conductive network.

**Figure 7:**
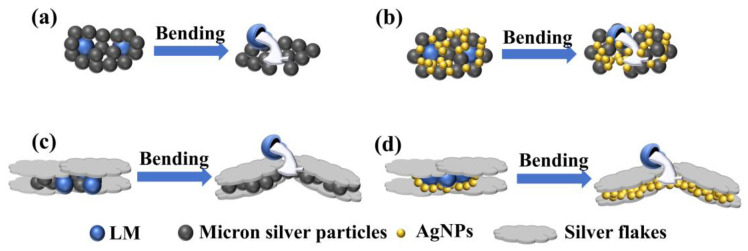
Bending mechanism diagram of conductive lines prepared by different conductive inks: (**a**) liquid metal/micron silver particles; (**b**) liquid metal/micron silver particles/silver nanoparticles; (**c**) liquid metal/micron silver particles/silver flakes; (**d**) liquid metals/silver nanoparticles/silver flakes.

Figure 7 illustrates the mechanism underlying the difference in bending resistance of conductive lines. When the conductive network contains only micron silver particles, microcracks form within the network after numerous bends, leading to a sharp decline in electrical conductivity. With the addition of liquid metal, external mechanical force causes the liquid metal to rupture, and the overflow fills the gaps between the ruptured micron silver particles, thereby enhancing particle contact and improving bending resistance (as shown in Figure 7a). Incorporating AgNPs into the network fills the voids and holes, increasing the number of contact points between adjacent conductive fillers when the liquid metal breaks. This enhances the bending resistance of the conductive lines (as depicted in Figure 7b,d). After adding silver flakes, the reduction in electrical conductivity during bending primarily arises from the slip and separation of the flakes. When AgNPs or micron silver particles are present in the network, they fill the gaps between adjacent silver flakes, improving the contact between them and enhancing the flexibility of the conductive lines. Additionally, due to their large surface area after hot pressing, silver flakes facilitate the rupture of liquid metal under mechanical strain, increase the number of ruptured liquid metal connections, and thereby improve the mechanical stability of the conductive network (as shown in Figure 7c,d).

**Figure 8:**
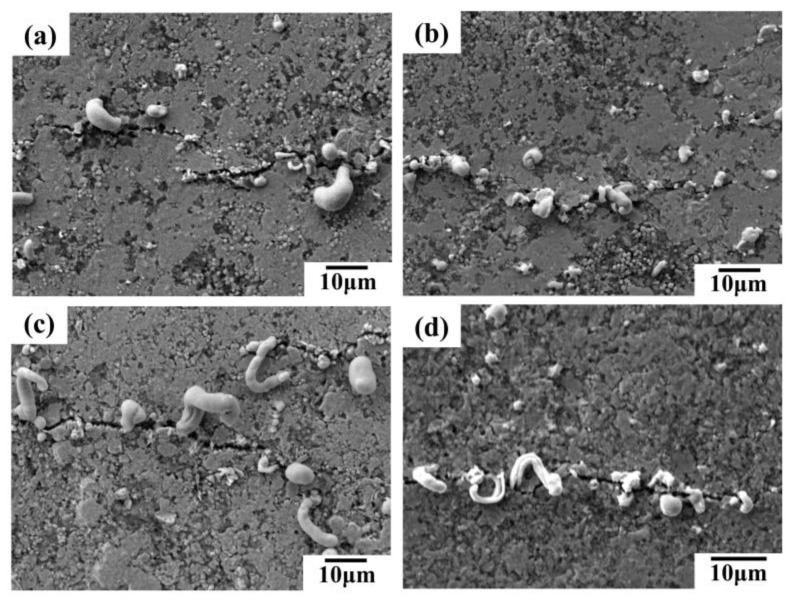
SEM images of conductive lines prepared by different conductive inks after 5000 bending cycles: (**a**) liquid metal/micron silver particles; (**b**) liquid metal/micron silver particles/silver nanoparticles; (**c**) liquid metal/micron silver particles/silver flakes; (**d**) liquid metal/silver nanoparticles/silver flakes.

The SEM images in Figure 8 reveal the surface morphology of conductive lines prepared with different inks after 5000 bending cycles. Microcracks, perpendicular to the bending direction, are observed on the surface of all the lines (Figure 8). These cracks contribute to the increased resistance shown in Figure 6a,b. The bending process likely induces liquid metal rupture within the conductive network, causing some metal to migrate towards the silver fillers and extrude at the crack sites. Notably, lines containing silver flakes exhibit more pronounced liquid metal extrusion compared to those without flakes, sometimes forming strips (Figure 8c,d). Additionally, lines with silver nanoparticles (AgNPs) show some AgNP extrusion at the cracks (Figure 8b,d), potentially acting as bridges and enhancing the network’s bending resistance. Consequently, conductive lines incorporating various silver fillers and liquid metal demonstrate superior mechanical stability and bending resistance compared to lines with only micron silver particles. Among these, the line formulated with liquid metal, micron silver particles, and silver flakes exhibits the best bending resistance.

### 3.2. Optimizing Conductive Ink Performance through the Mass Ratio of Fillers

**Figure 9:**
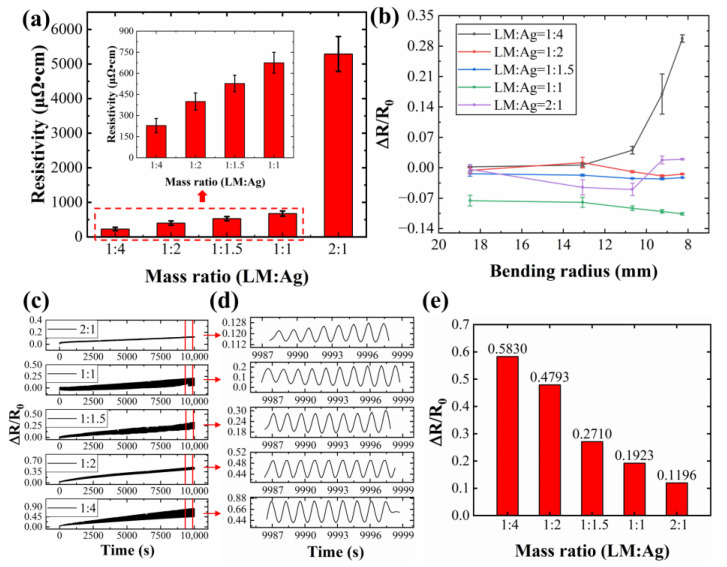
Electrical properties and mechanical stability of conductive lines prepared by conductive inks with different mass ratios of liquid metal to silver: (**a**) electrical properties; (**b**) resistance change rate at different bending radii; (**c**) resistance change rate during 5000 bending cycles; (**d**) magnification of the red area in figure (**c**); (**e**) resistance change rate after 5000 bending cycles.

Five conductive inks with varying liquid metal (LM) to silver mass ratios were prepared, and their corresponding conductive lines were tested (Figure 9). As shown in Figure 9a, the conductivity in LM/silver inks depends primarily on the silver network and fractured LM particles. A lower LM content translates to a higher silver fraction, leading to lower resistivity and improved electrical performance. For example, the conductive line exhibits a resistivity of 675.14 μΩ·cm at a 1:1 LM:silver ratio, which decreases to 228.77 μΩ·cm with a 1:4 ratio.

Bending tests were performed on conductive lines with varying liquid metal to silver mass ratios to investigate their resistance changes at different bending radii (Figure 9b). As shown in Figure 9b, only the conductive line with a 1:1 mass ratio exhibits a continuous decrease in resistance change rate with the increasing bending radius. The other lines show an initial decrease at small bending angles, followed by an increase beyond a certain point. At maximum bending, the 1:4 ratio line exhibits the highest resistance change rate, with a 29.74% increase.

As previously discussed, bending induces both network reconstruction and failure within the conductive lines. When reconstruction dominates (resistance decreases), the network maintains conductivity. Conversely, failure (resistance increases) weakens the network. High liquid metal or silver content weakens the reconstruction relative to failure. For a high liquid metal content, the network becomes more rigid, hindering reconstruction. A high silver content increases the probability of particle fracture and network disruption. These factors lead to increased resistance and decreased electrical conductivity at high bending degrees (Figure 9b).

Figure 9c–e illustrate the resistance change rate of conductive lines with different mass ratios over 5000 bending cycles. As evident in Figure 9c,d, a higher liquid metal content leads to smaller resistance changes during bending, indicating enhanced network stability. Additionally, a higher liquid metal content results in lower post-bending resistance (Figure 9d). Notably, the line with a 2:1 liquid metal to silver ratio exhibits only an 11.96% increase in resistance after 5000 bending cycles (Figure 9e).

**Figure 10:**
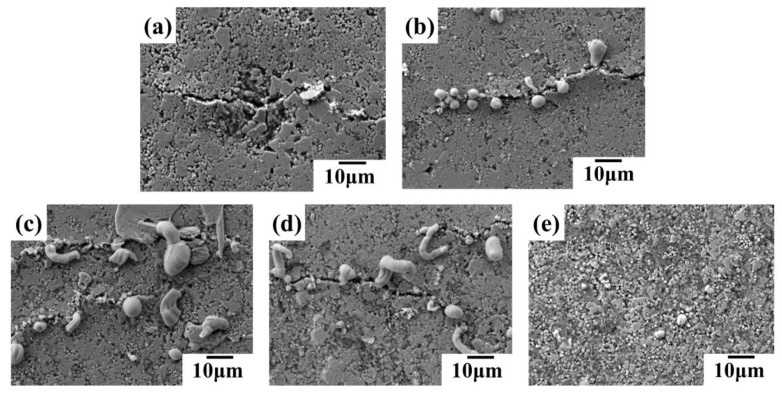
SEM images of conductive lines prepared by conductive inks with different mass ratios of liquid metal to silver after 5000 bending cycles: (**a**) 1:4; (**b**) 1:2; (**c**) 1:1.5; (**d**) 1:1; (**e**) 2:1 (liquid metal:silver).

The SEM images in Figure 10 reveal the influence of the liquid metal (LM) to silver mass ratio on the morphology of conductive lines after 5000 bending cycles. The line with a 1:4 LM:silver ratio (Figure 10a) exhibits microcracks without any LM extrusion at these cracks. As the LM content increases from 1:2 (Figure 10b) to 1:1 (Figure 10d), LM starts to appear at the microcracks, transitioning from droplets to strips, suggesting more pronounced LM extrusion. Interestingly, at a 2:1 LM:silver ratio (Figure 10e), the extruded LM reverts to a droplet shape, while the number of microcracks decreases. This behavior can be attributed to the higher LM content. Upon rupture of the LM oxide film, the numerous LM droplets merge, exhibiting self-healing properties and suppressing microcrack formation.

**Figure 11:**
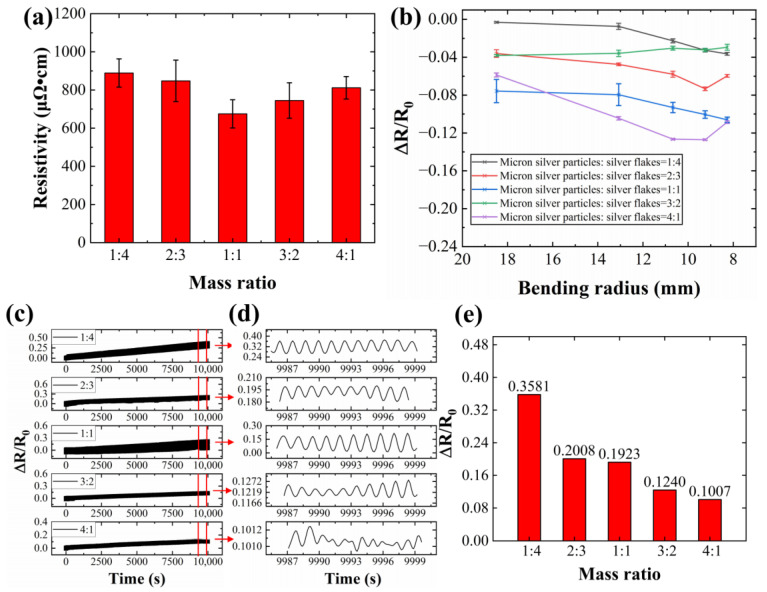
Electrical properties and bending resistance of conductive lines prepared by conductive inks with different mass ratios of micron silver particles to silver flakes: (**a**) electrical properties; (**b**) resistance change rate at different bending radii; (**c**) resistance change rate during 5000 bending cycles; (**d**) magnification of the red area in figure (**c**); (**e**) resistance change rate after 5000 bending cycles (micron silver particles:silver flakes).

We investigated the impact of the mass ratio between micron silver particles and silver flakes on conductive ink performance. Five conductive inks were formulated with varying mass ratios, and corresponding conductive lines were fabricated. The performance of these lines was evaluated after hot pressing treatment (Figure 11).

Figure 11a reveals an initial decrease in the conductive line’s resistivity, followed by an increase with the increasing silver flake mass fraction. The lowest resistivity of 675.14 μΩ·cm is achieved with a 1:1 mass ratio of micron silver particles to silver flakes. This initial improvement can be attributed to the increased number of contact points within the conductive network formed by the additional silver flakes. However, exceeding a 12.5% mass fraction of silver flakes leads to a decline in conductivity. This is likely due to the accumulation of flakes and the formation of voids between them, hindering electrical pathways.

**Figure 12:**
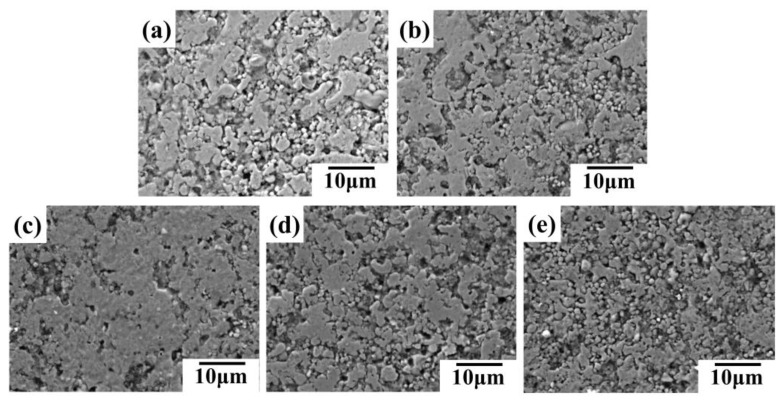
SEM images of conductive lines prepared by conductive inks with different mass ratios of micron silver particles to silver flakes after hot pressing: (**a**) 1:4; (**b**) 2:3; (**c**) 1:1; (**d**) 3:2; (**e**) 4:1 (micron silver particles:silver flakes).

The SEM images in Figure 12 support this observation. Figure 12a,b depict excessive silver flake contents leading to accumulation and void formation, resulting in a lower surface density of the conductive line and decreased electrical conductivity. Conversely, insufficient silver contents (Figure 12d,e) also lead to a less dense surface. Therefore, the silver flake content significantly affects the electrical conductivity of the conductive coating. An optimal balance is achieved at a 12.5% silver flake mass fraction, leading to a denser conductive line surface and maximized performance.

Figure 11b depicts the resistance changes of conductive lines with varying mass fractions of silver flakes (5%, 10%, 12.5%, 15%, and 20%) subjected to different bending radii. Lines with a lower silver flake content (5%) exhibit a sharp increase in resistance at higher bending angles, indicating a greater susceptibility to mechanical stress. Conversely, lines with a higher silver flake content (20%) show a more stable resistance profile under larger bending radii.

**Figure 13:**
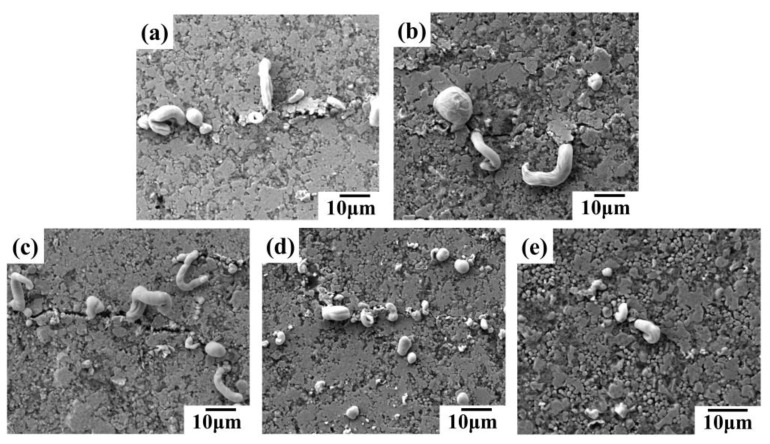
SEM images of conductive lines prepared by conductive inks with different mass ratios of micron silver particles to silver flakes after 5000 bending cycles: (**a**) 1:4; (**b**) 2:3; (**c**) 1:1; (**d**) 3:2; (**e**) 4:1 (micron silver particles:silver flakes).

Figure 11c–e illustrate the results of bending fatigue tests (5000 cycles) on conductive lines with different silver flake contents. The resistance of the lines generally decreases with the increasing silver content. This trend might be attributed to the vulnerability of silver flakes to mechanical stress, as evidenced by microcrack formation and disruption of the outer oxide film on the liquid metal particles (Figure 13). At a lower silver flake content (5%, Figure 13e), fewer microcracks are observed after bending cycles, suggesting better structural integrity. In contrast, samples with a high silver flake content (20%, Figure 13a) exhibit extensive microcracks and breakage of silver flakes, leading to a significant decrease in bending resistance.

### 3.3. Influence of Sintering Parameters on the Properties of Conductive Inks

A conductive ink was prepared using a filler composition of 25 wt% liquid metal (LM), 12.5 wt% micron silver particles, and 12.5 wt% silver flakes to study the influence of hot pressing parameters on the resistivity of conductive lines. Five samples were measured under each parameter to minimize errors.

**Figure 14:**
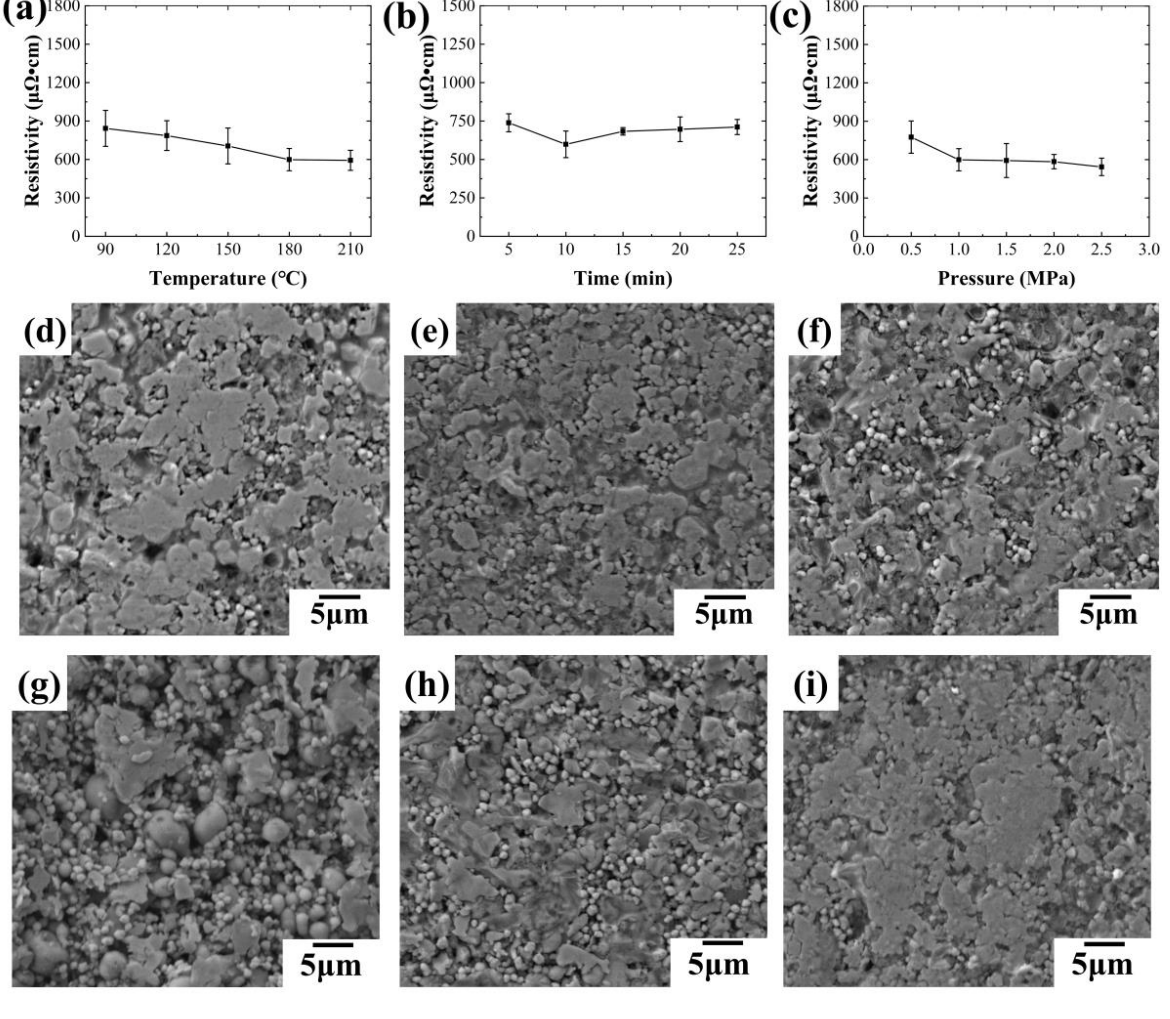
Effects of sintering parameters on the conductivity of conductive lines: (**a**) effects of sintering temperature on the conductivity of conductive lines; (**b**) effects of sintering time on the conductivity of conductive lines; (**c**) effects of sintering pressure on the conductivity of conductive lines; (**d**–**f**) SEM images of conductive lines at different sintering times: (**d**) 5 min; (**e**) 15 min; (**f**) 25 min; (**g**–**i**) SEM images of conductive lines at different sintering pressures: (**g**) 0.5 MPa; (**h**) 1.5 MPa; (**i**) 2.5 MPa.

The effects of sintering temperature (10 min, 1.0 MPa) and time (180 °C, 1.0 MPa) were investigated first, as shown in Figure 14. Figure 14a depicts the impact of varying sintering temperatures on the resistivity of conductive lines. The results demonstrate a decrease in resistivity with the increasing temperature, likely due to the gradual decomposition of solvents and organic compounds within the ink, leading to improved conductivity. Notably, the decreasing trend plateaus at 180 °C, suggesting a diminishing effect of temperature on resistivity at higher values.

Figure 14b illustrates the effect of different sintering times on the resistivity of conductive lines during hot pressing sintering. As shown in the figure, the resistivity of the conductive line decreases with increasing the sintering time up to 10 min. However, after 10 min, the resistivity begins to increase with further increases in the sintering time, leading to a decrease in electrical conductivity. To investigate this behavior, conductive lines treated with various sintering times were characterized microscopically, and SEM images are presented in Figure 14d–f. After hot pressing, the oxide layer of the liquid metal on the surface of the conductive line breaks under pressure, causing the internal liquid metal to flow out and fill the holes in the surface layer. Additionally, silver flakes adhere to the surface of the conductive coating, resulting in a smoother surface for the conductive line. A smoother surface typically indicates better conductivity. From Figure 14d–f, it can be observed that, with a sintering time of 5 min, the surface of the conductive line is smooth, resulting in an improved conductive performance. However, as the sintering time increases to 15 min, the surface of the conductive line becomes rougher, leading to a decline in electrical conductivity. Further increasing the sintering time to 25 min results in an even rougher surface of the conductive line, further increasing the resistivity.

Additionally, to explore the effect of different sintering pressures on the electrical conductivity of the conductive line, the line was sintered under varying pressures of 0.5 MPa, 1.0 MPa, 1.5 MPa, 2.0 MPa, and 2.5 MPa at a sintering temperature of 180 °C and for a duration of 10 min. Figure 14 illustrates the impact of sintering pressure on the resistivity of the conductive lines. As shown in Figure 14c, the resistivity of the conductive line decreases with the increasing sintering pressure. Higher sintering pressures result in better conductivity of the conductive line. Figure 14g–i displays the micromorphology of conductive lines under different sintering pressures. At a low sintering pressure (0.5 MPa), the surface of the conductive line is uneven, with numerous holes visible. Additionally, many liquid metal particles remain unbroken, with silver flakes adhering to the outer surface of these particles, significantly affecting the electrical conductivity of the line. With an increase in pressure, more liquid metal ruptures inside the conductive line, leading to a smoother surface and fewer holes in the conductive line. This reduction in surface defects contributes to the decreased resistivity of the conductive line.

To further investigate the effect of sintering process parameters on the conductive line of liquid metal:micron silver conductive ink, a set of orthogonal experiments was designed using liquid metal, micron silver particles, and silver flakes at a mass ratio of 2:1:1, as detailed in Table 1. The orthogonal experiment included three factors with four levels each. The factors were temperature, time, and pressure. Four levels for each were used in the experiment, with a total of 16 groups of experiments. According to the designed sintering process parameters, the conductive lines were hot-pressed and sintered, and then, the resistance was measured. Each measurement was averaged over 10 trials to reduce experimental error. The resistivity of the conductive lines under different sintering process parameters is summarized in Table 1.

After obtaining the test results in Table 1, the range analysis method was employed to analyze the experimental data, and the results are summarized in Table 2. In Table 2, $K_{I}$ represents the sum of the experiments of this factor in the case of horizontal I; $\bar{K_{I}}$ represents the mean of $K_{I}$, which is used to calculate the R value; R is the range value, which is obtained by the maximum mean of $K_{I}$ minus the minimum mean of $K_{I}$, and the primary and secondary order of factors can be sorted according to the size of the R value. The number of repetitions per level of factors represents the average number of experiments conducted horizontally. Table 2 shows that the sintering temperature has a greater effect on the resistivity of conductive lines compared to the sintering pressure and sintering time.

### 3.4. Demonstration of Flexible Wire-Conductive Polymer Integrated Unit

**Figure 15:**
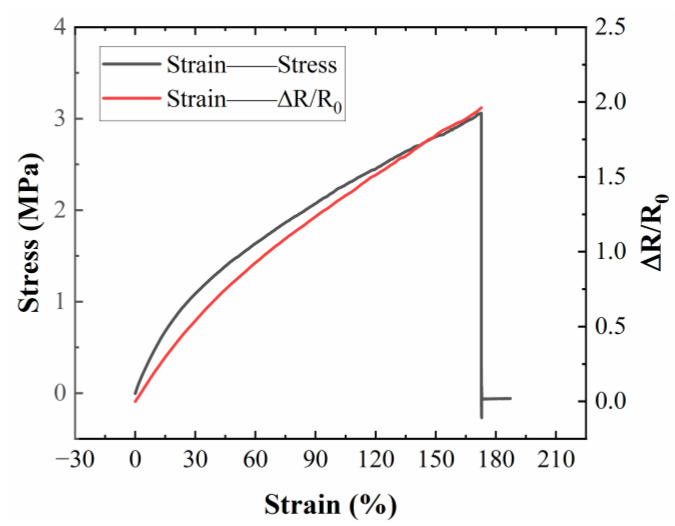
Mechanical–electrical properties of conductive polymers.

We selected conductive inks comprising 25% liquid metal, 12.5% micron silver particles, and 12.5% silver flakes to fabricate flexible wires. Subsequently, we utilized conductive polymers containing 20% conductive fillers, 2.5% carbon nanotubes, and 22.5% silver flakes as flexible sensors. The mechanical–electrical properties of the conductive polymer are illustrated in Figure 15, demonstrating an elongation at the break of up to 170% and excellent tensile properties. Additionally, the conductive polymer exhibits outstanding strain sensing performance and sensitivity under both small and large strains.

**Figure 16:**
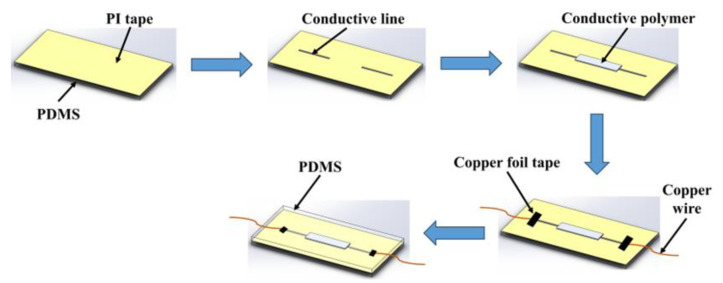
Schematic diagram of the preparation process of the flexible wire-flexible sensor integrated unit.

**Figure 17:**
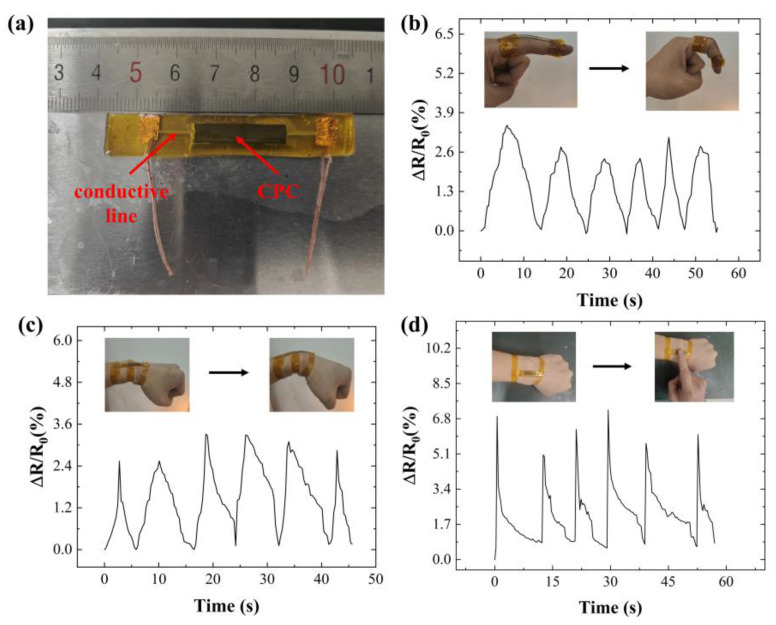
Flexible wire-conductive polymer integrated unit application testing: (**a**) flexible wire-conductive polymer integrated unit; (**b**) finger bending detection; (**c**) wrist bending detection; (**d**) compression test.

The fabrication process of the flexible wire-flexible sensor integrated unit is illustrated in Figure 16. First, a layer of PI tape (thickness 55 μm) is affixed to the PDMS substrate. Then, the flexible wire is prepared on the PI layer using conductive ink. Once the flexible wire is prepared, conductive silver paste is applied to both ends of the conductive polymer to connect the flexible wire and the conductive polymer. After the silver paste has fully cured, copper wire is connected to both ends of the wire. Finally, a layer of PDMS is coated over the PI film, and the packaging layer is formed after heating and curing. The integrated unit of conductive wire and CPC-based strain sensor for human motion detection is presented in Figure 17a. Particularly, the length, width, and thickness of the conductive wire are 30 mm, 1 mm, and 55 μm, respectively. The length, width, and thickness of the conductive polymer are 25 mm, 5 mm, and 0.5 mm, respectively.

The integrated unit was affixed to the experimenter’s finger and wrist joints using PI tape to measure the bending angles. A change in resistance was detected when the finger or wrist was bent, as depicted in Figure 17b,c. During finger and wrist bending, the resistance of the integrated unit changed gradually, indicating excellent mechanical properties of the flexible wire prepared with conductive ink and reliable integration with the conductive polymer. Furthermore, the integrated unit was capable of detecting small pressing actions, as illustrated in Figure 17d.

## 4. Conclusions

Our study successfully developed liquid metal:silver conductive inks utilizing liquid metal and two distinct silver filler morphologies (particles and flakes). It was found that lines formulated with a combination of all three conductive agents exhibited the highest conductivity without post-treatment. Additionally, post-treatment effectively enhanced their mechanical properties, resulting in a mere 19.23% increase in resistance after 5000 bending cycles. Additionally, we investigated the influence of the mass ratio of conductive fillers on the composite ink’s properties. Increasing the liquid metal content yielded a trade-off, decreasing conductivity while bolstering mechanical stability. The optimal conductivity was achieved with a 1:1 mass ratio of micron silver particles to silver flakes, while a 4:1 ratio offered superior mechanical performance (resistance increase of only 10.07% after 5000 bending cycles). However, a higher silver flake content compromised the mechanical stability. Furthermore, we evaluated the impact of hot pressing sintering parameters on the conductive lines’ properties. Both conductivity and sintering temperature/pressure exhibited a positive correlation. Sintering time also influenced conductivity, initially increasing up to 10 min before declining with further extension. Particularly, sintering temperature exerted the most significant effect on conductivity, followed by pressure and then time.

To demonstrate the practical application, we successfully fabricated flexible wires by integrating the composite conductive ink with conductive polymers. This integrated system effectively detected various human body movements, including finger and wrist bending, as well as finger pressing. These findings significantly strengthen the potential of liquid metal:silver-filled conductive inks for robust and durable circuit applications.

## Figures and Tables

**Table 1:** Resistivity measurement results of different experimental schemes.

Serial Number	Temperature (°C)	Time (min)	Pressure (MPa)	Resistivity (μΩ·cm)
1	120	5	0	1040.81
2	120	10	0.5	879.48
3	120	15	1.5	1061.34
4	120	20	2.5	528.37
5	150	5	0.5	657.53
6	150	10	0	919.38
7	150	15	2.5	592.40
8	150	20	1.5	813.33
9	180	5	1.5	621.39
10	180	10	2.5	500.28
11	180	15	0	537.26
12	180	20	0.5	777.39
13	210	5	2.5	701.27
14	210	10	1.5	669.78
15	210	15	0.5	550.30
16	210	5	0	710.45

**Table 2:** Results of orthogonal experiments for the sintering process parameters.

Item	Temperature	Time	Pressure
$K_{1}$	3510.00	3021.00	3207.91
$K_{2}$	2982.63	2968.92	2864.70
$K_{3}$	2436.32	2741.30	3165.83
$K_{4}$	2631.80	2829.54	2322.32
$\bar{K_{1}}$	877.50	755.25	801.98
$\bar{K_{2}}$	745.66	752.23	716.17
$\bar{K_{3}}$	609.08	685.32	791.46
$\bar{K_{4}}$	657.95	707.39	580.58
R	268.42	69.93	221.40
Repetition of experiment per level of factor	4	4	4
Primary and secondary order of factors	Temperature—Pressure—Time		

## Data Availability

Data are contained within the article.
